# Early Tranexamic Acid in Intracerebral Hemorrhage: A Meta-Analysis of Randomized Controlled Trials

**DOI:** 10.3389/fneur.2021.721125

**Published:** 2021-12-06

**Authors:** Xu Jiao, Mingfei Li, Lulu Li, Xinyu Hu, Xiaohui Guo, Yun Lu

**Affiliations:** ^1^Emergency Department, Hospital of Chengdu University of Traditional Chinese Medicine, Chengdu, China; ^2^Clinical Medical School, Chengdu University of Traditional Chinese Medicine, Chengdu, China

**Keywords:** tranexamic acid, intracranial hemorrhage, hematoma expansion, mortality, meta-analysis

## Abstract

**Objective:** Intracranial hemorrhage (ICH) is a common complication of traumatic brain, in which tranexamic acid has been recommended as an additional therapy to prevent a second bleeding. However, the effect of early administration of tranexamic acid for ICH patients remains controversial.

**Methods:** A systematic search was performed in Cochrane Library, Medline, Embase, and Web of Science. Poor outcome refers to significant hemorrhage growth, new intracranial hemorrhage, new focal cerebral ischaemic lesions, the need for neurosurgery, or death. Study heterogeneity and publication bias were estimated.

**Results:** Seven randomized controlled trials involving 3,192 participants were included in our meta-analysis. Tranexamic acid administration in ICH patients was associated with better outcomes of hematoma expansion (odd ratios [OR] 0.79; 95% confidence interval (CI) CI, 0.67–0.93; *I*^2^ = 0%; *P* = 0.006) and growth of hemorrhagic lesions (weighted mean difference [WMD], −1.97 ml; 95% CI, −2.94 to −1.00; *I*^2^ = 14%; *P* < 0.001) than the placebo. No difference was found between the mortality, poor outcome, neurosurgical intervention, new bleeding, and the duration of hospital stay. Moreover, no publication bias was found.

**Conclusion:** Our analysis reveals that the early treatment with tranexamic acid can significantly reduce the incidence of hematoma expansion and the volume of hemorrhagic lesion, but does not exert considerable effects on mortality, poor outcome, neurosurgery, rebleeding, and the duration of stay.

## Introduction

With more than 10 million hospitalized patients and ~1.5 million fatal cases, intracerebral hemorrhage (ICH) is one of most common and lethal complications of traumatic brain injury in the United States and Japan (1, 2). ICH leads to coagulopathy and hemorrhage expansion, resulting in high disability, and mortality (3). Hemorrhage expansion is a well-known key factor for unfavorable ICH outcomes (4). Although an early reduction in the intensive blood pressure was reported to improve the functional outcome, treatment in ICH patients did not contribute to a significant improvement in survival (5, 6).

Antifibrinolytic treatment has been recommended as an additional therapy to prevent a second bleeding by increased fibrinolysis and augmented levels of fibrin degradation products after ICH (7). Tranexamic acid is a potent antifibrinolytic drug that has been widely used in menorrhagia and cardiac surgery (8, 9). It was reported to block the lysine-binding sites on plasminogen molecules, significantly reducing the morality from 4.9 to 5.7% in trauma patients (10). However, the frequency of death and adverse outcome after ICH was not improved after tranexamic acid treatment, even if it had a significant effect on the control of hemorrhage expansion (11). In previous studies, a treatment with this drug was applied 8 h after symptom onset of hyperacute intracerebral hemorrhage (12, 13).

In the CRASH-2 trial, trauma patients were treated with tranexamic acid within 8 h after the onset, based on the fact that most bleeding fatalities occur on the day of the injury. This investigation evidenced that the timely administration of tranexamic acid was essential to its beneficial effect in patients (10). However, to date, the influence of early administration of tranexamic acid in ICH patients remains unknown. Therefore, to provide high-quality evidence for further clinical experiments, we conducted a meta-analysis of the randomized controlled trials on the early administration of tranexamic acid in the treatment of ICH patients.

## Methods

We performed the study in adherence to the PRISMA (Preferred Reporting Items for Systematic Reviews and Meta-Analysis) guidelines (14). The protocol has been registered in the PROSPERO international prospective register of systematic reviews.

### Search Strategy

We comprehensively searched for articles indexed in Cochrane Central, Medline, Embase, and Web of Science up to 23 December 2020. The studies for inclusion were limited to reports of adult human trials, with no language, or other limits applied. The following key words were used in the search: “tranexamic acid,” “cerebral hemorrhage OR intracranial hemorrhage OR subarachnoid hemorrhage OR hemorrhage.” In addition, we examined the reference lists of the included articles for additional studies.

### Selection and Exclusion Criteria

Studies meeting the following criteria were eligible for inclusion: (1) all patients were diagnosed with cerebral hemorrhage; (2) a randomized controlled trial (RCT); and (3) the patients were treated with tranexamic acid or placebo within 8 h post onset (12). The exclusion criteria were as follows: (1) studies that did not contain the outcomes of interest, such as hematoma expansion, volume of the hemorrhagic lesion, mortality, poor outcome, neurosurgical intervention, new bleeding, or hospital stay; (2) reviews, case reports, or series and comments; (3) repeated samples were enrolled in the studies; and (4) the studies were based on animal experiments.

### Data Extraction and Quality Assessment

Rigorous data collection was performed independently by two authors (XJ and ML) for data extraction. Differences in opinions were resolved by reaching a consensus with a third author. The main characteristics of the eligible studies included the first author's name, publication year, country, sample size, gender, and age of each group. Two authors independently assessed the quality of the studies included in this systematic review using the Jadad scale (15). The scores of Jadad scale range from 0 to 7, and studies with a score above 3 were considered to be of high quality.

### Statistical Analysis

Odds ratios (OR) with 95% confidence intervals (CI) were used to pool the dichotomous variables, and the weighted mean difference (WMD) was utilized to assess the continuous variables. *I*^2^-test was used to assess statistical heterogeneity. For outcomes with low heterogeneity (*I*^2^ < 50% and *P* > 0.1), a fixed-effects model (the Mantel–Haenszel method) was employed for secondary analysis; otherwise (*I*^2^ ≥ 50% or *P* ≤ 0.1), a random-effect model (the DerSimonian and Laird method) was adopted (16). Publication bias was evaluated via visual analysis of funnel plots and Egger's and Begg's tests (17, 18). Sensitivity analysis was further conducted, in which one study was removed and the rest were analyzed to evaluate the statistical significance of the impact on the results. Thus, sensitivity analysis was performed to assess the potential sources of heterogeneity in the analyses. All tests were considered to indicate statistically significant differences at *P* < 0.05. All statistical analyses were conducted using Review Manager 5.3 (Cochrane Collaboration, UK) and STATA Version 14.0 (Stata Corporation LP, College Station, TX, USA).

## Results

### Study Selection and Description

**Figure 1:**
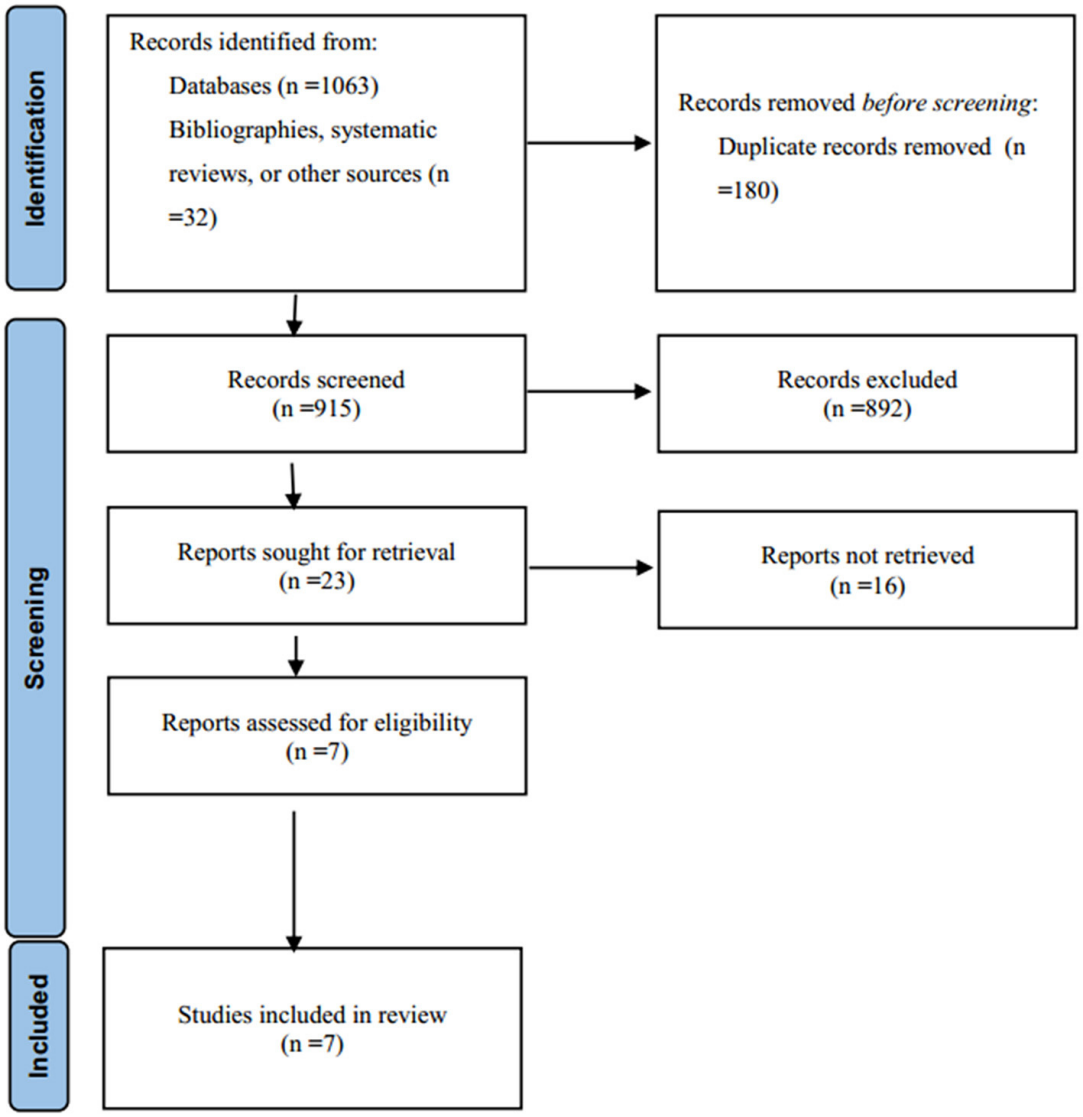
Flowchart of the study selection process for inclusion in the meta-analysis.

The search strategy identified 1,063 studies and 32 records from bibliographies and systematic reviews. A number of 180 studies were excluded for duplications. After reviewing the title and abstract, we excluded 892 articles following the exclusion criteria. Sixteen studies were excluded after assessing their full texts; the patients in 12 studies were treated with tranexamic acid or placebo later than 8 h after onset, 2 articles did not include the outcome of interest, and 2 studies did not have a control group. Of note, the CRASH-3 trial (19) included a large number of TBI patients who were treated by tranexamic acid within 3 h, however, not all included patients developed intracranial bleeding, so this trial was not included for analysis. Ultimately, seven randomized controlled trials were included in this meta-analysis (11, 12, 20–24) (Figure 1). Based on the Jadad scale range for the assessment of the quality of the studies, all of the included six studies were rated with a total score of >3, indicating that they were high-quality studies (Supplementary Table 1). A number of 3,192 patients with ICH were enrolled in these trials; 1,593 patients were treated with tranexamic acid and 1,599 patients with placebo. The main characteristics of the included trials are presented in Table 1.

**Table 1:** Characteristics of the included studies.

**Author**	**Year**	**Country**	**ICH type**	**No. of patients**		**Male/Female**		**Age (years)**		**Jadad score**
				**TA**	**PC**	**TA**	**PC**	**TA**	**PC**	
Crash-2 (11)	2011	India and Colombia	Traumatic ICH	133	137	111/22	117/18	36 ± 14	37 ± 14	7
Yutthakasemsunt et al. (20)	2013	Thailand	Traumatic ICH	120	118	103/17	107/11	34.8 ± 16.0	34.1 ± 15.3	4
Arumugam et al. (21)	2015	Malaysia	Hypertensive ICH	15	15	NR	NR	> 18	> 18	5
Fakharian et al. (22)	2017	Iran	Traumatic ICH	74	75	67/7	66/9	42.8 ± 18.3	39.3 ± 18.1	4
Jokar et al. (23)	2017	Iran	Acute traumatic ICH	40	40	32/8	28/12	35.4 ± 14.6	36.2 ± 14.9	4
Sprigg et al. (12)	2018	12 countries	Primary ICH	1,161	1,164	642/519	659/505	69.1 ± 13.7	68.7 ± 13.9	7
Meretoja et al. (24)	2020	3 countries	Acute ICH	50	50	35/15	27/23	71 (58–79)	73 (55–78)	7

*NR, non-reported; ICH, intracerebral hemorrhage; TA, tranexamic acid; PC, placebo control*.

### Hematoma Expansion

**Figure 2:**
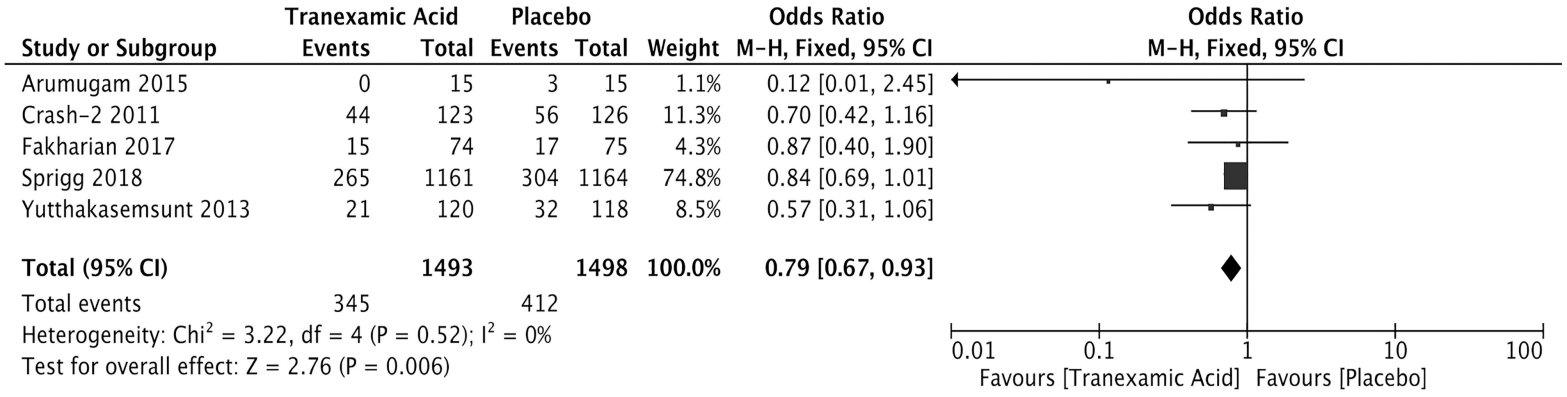
Forest plot of the comparison between tranexamic acid and placebo treatments for hematoma expansion.

The measurement of hematoma expansion in most trials was detected by computed tomography (Supplementary Table 2). We calculated the pooled OR of 2,991 patients with available data for hematoma expansion. The incidence rate of hematoma expansion was significantly lower in the tranexamic acid group than in the placebo group (OR = 0.79; 95% CI, 0.67–0.93; *I*^2^ = 0%; *P* = 0.006) (Figure 2).

### Volume of the Hemorrhagic Lesion

**Figure 3:**
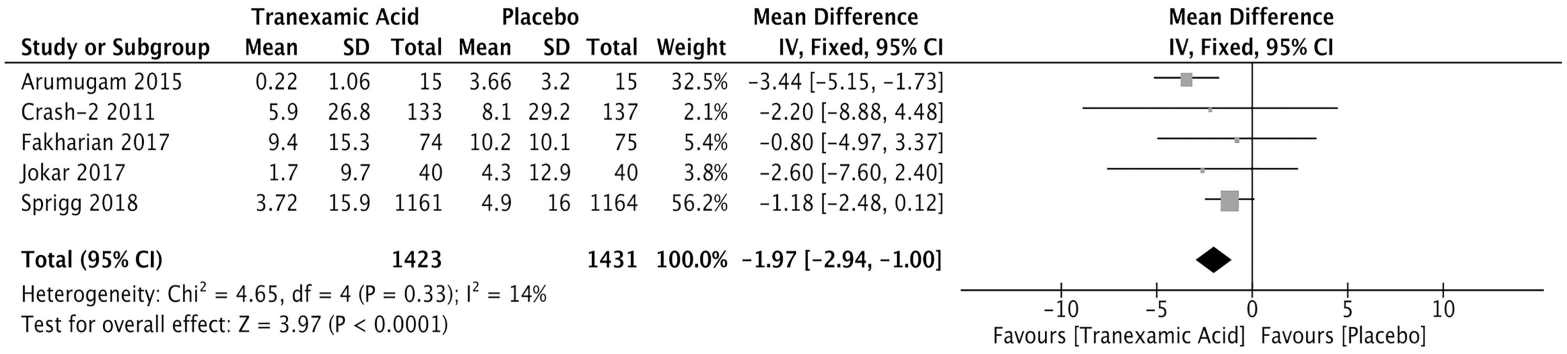
Forest plot of the comparison between tranexamic acid and placebo treatments for the volume of hemorrhagic lesion.

Five studies enrolling 2,854 patients reported the volume of the hemorrhagic lesion. The hemorrhagic lesion was detected 24 h after treatment (Supplementary Table 3). The pooled weighted mean difference (WMD) for the volume (ml) of the hemorrhagic lesion was −1.97 (95% CI: −2.94 to −1.00, *I*^2^ = 14%; *P* < 0.001), indicating that the volume of the hemorrhagic lesion was significantly lower in the patients treated with tranexamic acid than in those in the control group (Figure 3).

### Mortality

**Figure 4:**
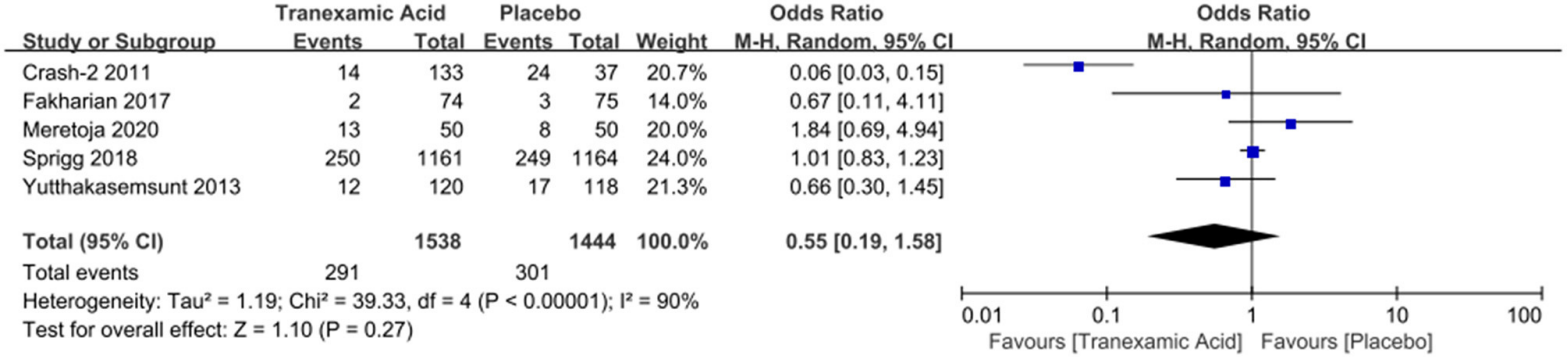
Forest plot of the comparison between tranexamic acid and placebo treatments for the mortality.

Four studies with a total number of 2,882 patients investigated the correlation between early tranexamic acid and mortality. The pooled OR for mortality after hemorrhage was 0.55 (95% CI: 0.19–1.58, *I*^2^ = 90%; *P* = 0.27), revealing that the early treatment with tranexamic acid did not decrease significantly the mortality of ICH patients (Figure 4).

### Poor Outcome

**Figure 5:**
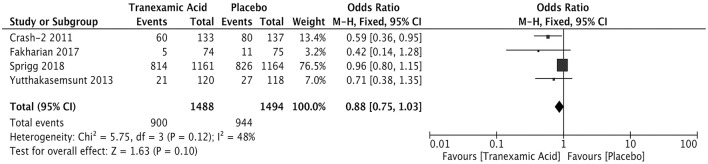
Forest plot of the comparison between tranexamic acid and placebo treatments for the poor outcome.

Four studies involving 2,982 patients analyzed the relationship between the early treatment with tranexamic and poor outcome after ICH. The results showed comparable rates of poor outcome (OR = 0.88; 95% CI: 0.75–1.03, *I*^2^ = 48%; *P* = 0.10) (Figure 5).

### Neurosurgical Intervention

**Figure 6:**
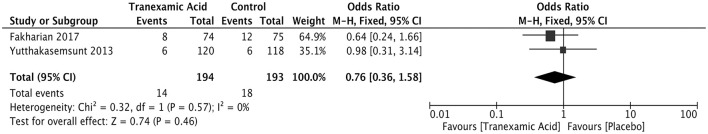
Forest plot of the comparison between tranexamic acid and placebo treatments for neurosurgical intervention.

Neurosurgical intervention was reported in two studies after early treatment with tranexamic acid of ICH patients. No difference was observed between the two groups of the ICH patients in terms of neurosurgical intervention (OR = 0.76; 95% CI: 0.36–1.58, *I*^2^ = 0%; *P* = 0.46) (Figure 6).

### New Bleeding

**Figure 7:**
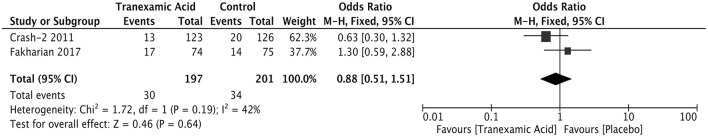
Forest plot of the comparison between tranexamic acid and placebo treatments for new bleeding.

The pooled ORs for the incidence rate of new bleeding of 298 patients from two trials was OR = 0.88 (95% CI, 0.51–1.51; *I*^2^ = 42%; *P* = 0.64) (Figure 7).

### Hospital Stay

**Figure 8:**
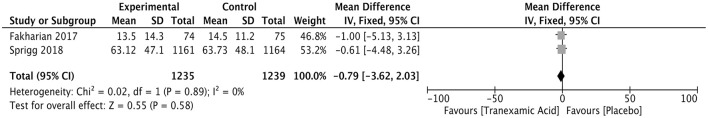
Forest plot of the comparison between tranexamic acid and placebo treatments for the duration of hospital stay.

The duration of the hospital stay was compared between a tranexamic acid-treated and a control group of ICH patients in two studies with 2,474 patients. The WMD was −0.79 (95% CI, −3.62 to 2.03; *I*^2^ = 0%; *P* = 0.58), showing no difference in the duration of hospital between the two groups (Figure 8).

### Publication Bias

The Egger's and Begg's tests provided no evidence of publication bias for the incidence rate of hematoma expansion (*P* = 0.1 and *P* = 0.221, respectively) and the volume of the hemorrhagic lesion (*P* = 0.218 and *P* = 0.08, correspondingly). In addition, the funnel plot for the incidence rate of the hematoma expansion (Supplementary Figure 1) and the volume of the hemorrhagic lesion (Supplementary Figure 2) appeared generally symmetrical.

### Sensitivity Analysis

In the sensitivity analyses, in which one study was excluded at a time from each analysis, the summary estimates were not substantially altered for the hematoma expansion (Supplementary Figure 3) and mortality (Supplementary Figure 4), whereas changes were present in the volume of the hemorrhagic lesion (Supplementary Figure 5).

## Discussion

The results from our meta-analysis of seven randomized controlled trials provide evidence in support that the ICH patients treated with tranexamic acid at 8 h had a lower incidence rate of hematoma expansion and a smaller volume of ICH than those in the control group. However, the early application of tranexamic acid did not reduce mortality. Furthermore, there were no differences in the poor outcome, neurosurgical intervention, rebleeding, and the duration of hospital stay between the tranexamic acid-treated and the placebo groups.

It is noteworthy that the lower incidence rate of hematoma expansion and the smaller volume of the hemorrhagic lesion in tranexamic acid-treated ICH patients did not reduce the mortality. Several possible explanations exist for this finding. First, it is important to note that tranexamic acid may increase the risk of cerebral ischemia in ICH and TBI as suggested by Roos et al. (25). Hence, the benefit was counteracted by an increase in the unfavorable outcome caused by cerebral ischemia as a result of the application of antifibrinolytics. Unfortunately, few included trials reported this outcome, and thus we could not properly analyze the effect of cerebral ischemia caused by tranexamic acid on ICH patients. Second, the population included in the present study was not sufficiently large.

In a study included more than 4,000 patients, a significant reduction in death caused by traumatic bleeding was found after early administration of tranexamic acid (26). In addition, more pronounced beneficial effects on patients are exerted if the administration of tranexamic acid is performed in <3 h after onset. Moreover, it was suggested that the survival benefit decreased by 10% for every 15 min of treatment delay until 3 h, after which there was no benefit for patients with acute severe hemorrhage (26). In this regard, early (within 3 h) administration with tranexamic acid may be recommended, which would result in a significant reduction of mortality. It should be noted that the time for death assessment was not always the same in each trial, for instance, in the study of Sprigg et al., early death was reduced, but not mortality at the end of the trial (12). Therefore, the conclusion for tranexamic acid in ICH treatment should be cautious.

In our study, the poor outcomes in the two groups were similar. Neurological surgery is one of the treatment strategies for patients with ICH, and a portion of ICH patients (~15%) undergo neurological intervention after the application of tranexamic acid (10). Notably, there was no increase in neurosurgical intervention for TA-treated patients. In the CRASH-2 trial, the ratio of patients undergoing surgery was equal in the two groups, which is in agreement with our results. A previous study on patients with aneurysmal subarachnoid hemorrhage reported that tranexamic acid reduced the rate of rebleeding. Nonetheless, we could not ignore the possibility that the doses of tranexamic acid applied in those studies were larger and the treatment periods more prolonged than in the included here studies (25). Furthermore, tranexamic acid exerted no effect on the length of hospital stay, probably because no beneficial effects were observed in the reduction in hematoma expansion.

To the best of our knowledge, this is the first meta-analysis focused on early administration of tranexamic acid in ICH patients, which revealed no effect of early administration of tranexamic acid on mortality. Two previous meta-analyses of the included studies were focused on the application of tranexamic acid in ICH patients, but not on early administration, which was quite different from the aims of our study. They obtained the same conclusion that the treatment with tranexamic acid did not improve the mortality and the poor functional outcomes but significantly decreased the growth of the hemorrhagic mass in ICH patients, reducing the clinical value of its widespread use (27, 28). However, a meta-analysis confirmed that tranexamic acid significantly diminished the death caused by acute severe bleeding (26). Depending on the individual participant condition, even a short treatment delay reduces the survival benefit from tranexamic acid for patients with acute severe bleeding, whereas the treatment without delay improved their survival by more than 70% (26). Several potential prerequisites exist that determine the requirement for the immediate treatment of ICH patients. Most importantly, fibrinogen stores are protected by the early application of tranexamic acid, leading to the formation of a stable fibrin clot. Since most deaths occur in the first few hours after bleeding onset, the beneficial effect on lowering mortality would be more pronounced if the lag between the onset and the administration of tranexamic acid can be maximally shortened. Meanwhile, more research is needed on the mechanisms of tranexamic acid on the mortality of such patients.

We acknowledge several limitations of our study. Significant heterogeneity existed on the mortality of patients, which might have limited the strength of our conclusion. In spite of the detailed sensitivity analyses performed, the differences in the application methods, the causes and types of cerebral hemorrhage, the treatment administration times, and disease severities could have been underestimated. Furthermore, the sample numbers from one of the trials represent more than 50% of all the involved patients in our analysis, increasing the risk of an overrepresentation. Additionally, the sample size was relatively small, compared with those of other meta-analyses on tranexamic acid treatment. Moreover, the included trials did not differentiate the types of ICH, intrcranial hemorrhage and spontaneous ICH were pooled together, which may introduce bias, future studies are warranted to separate intrcranial hemorrhage and spontaneous ICH as main outcomes.

## Conclusion

Our meta-analysis of six randomized controlled trials showed that the early treatment with tranexamic acid can significantly reduce the incidence of hematoma expansion and the volume of the hemorrhagic lesion in patients with ICH, but does not exert a considerable effect on the mortality, poor outcome, neurosurgery, rebleeding, and the duration of stay. In the future, high-quality RCTs with large sample sizes are needed to further explore the effect of early administration (e.g., within 3 h) tranexamic acid in ICH patients, which will require collaboration among surgeons, statisticians, neurologists, stroke physicians, geriatricians, trialists and patients.

## Data Availability Statement

The original contributions presented in the study are included in the article/Supplementary Material, further inquiries can be directed to the corresponding author/s.

## Author Contributions

XJ conceived and coordinated the study, designed, performed, and analyzed the data, and wrote the paper. ML, LL, and XH carried out the data collection and data analysis. XG and YL revised the paper. All authors reviewed the results and approved the final version of the manuscript.

## Funding

This study was supported by Science & Technology Department of Sichuan Province (Grant No. 2019YFS0040).

## Conflict of Interest

The authors declare that the research was conducted in the absence of any commercial or financial relationships that could be construed as a potential conflict of interest.

## Publisher's Note

All claims expressed in this article are solely those of the authors and do not necessarily represent those of their affiliated organizations, or those of the publisher, the editors and the reviewers. Any product that may be evaluated in this article, or claim that may be made by its manufacturer, is not guaranteed or endorsed by the publisher.
