# Supplementary material for: Early Tranexamic Acid in Intracerebral Hemorrhage: A Meta-Analysis of Randomized Controlled Trials
Source: Front Neurol. 2021 Dec 6;12:721125. doi: 10.3389/fneur.2021.721125 (PMC8685213; doi:10.3389/fneur.2021.721125)
Supplement: Supplementary file 1 [file Data_Sheet_1.pdf]

Table S1. Quality of the included studies.

Study	Randomization	Concealment of allocation	Double blinding	Withdrawals and dropouts	Jadad score
Crash-2 2011	2	2	2	1	7
Yutthakasemsunt et al. 2013	1	1	1	1	4
Arumugam et al. 2015	2	1	1	1	5
Fakharian et al. 2017	1	1	1	1	4
Jokar et al. 2017	1	1	1	1	4
Sprigg et al. 2018	2	2	2	1	7
Meretoja et al 2020	2	2	2	1	7

Table S2. Definition or measurement of haemorrhagic expansion.

Trials	Definition or measurement of haemorrhagic expansion
Crash-2	Difference in the combined volume (mL) of all intracranial haemorrhagic lesions from the first to the second CT scan.
Sprigg	Absolute increase of more than 6 mL or a relative growth of greater than 33%, detected by CT scan
Yutthakasemsunt	CT scan
Arumugam	CT scan
Fakharian	Not defined

CT, computed tomography.

Table S3. Timing for haemorrhagic lesion measurement.

Trials	Timing
Crash-2	Difference in the volume of all intracranial haemorrhagic lesions from the first to the second CT scan, specific timing not defined.
Sprigg	After 24 h of treatment
Yutthakasemsunt	24 hours ± 8 hours after the first CT scan
Arumugam	After 24 h of treatment
Fakharian	Within 24-48 hours

CT, computed tomography.

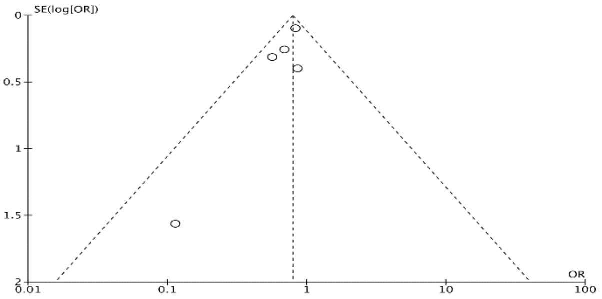

Figure S1. Funnel plot of the publication bias of hematoma expansion.

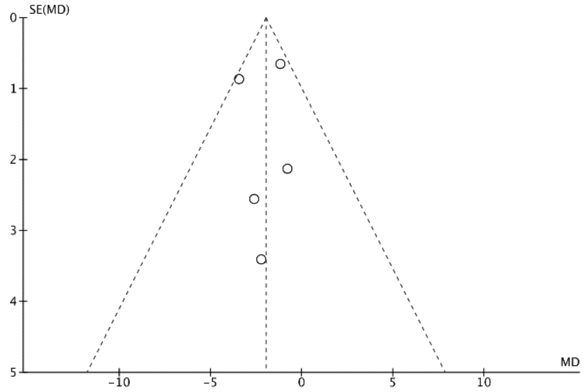

Figure S2. Funnel plot of the publication bias of the volume of the hemorrhagic lesion.

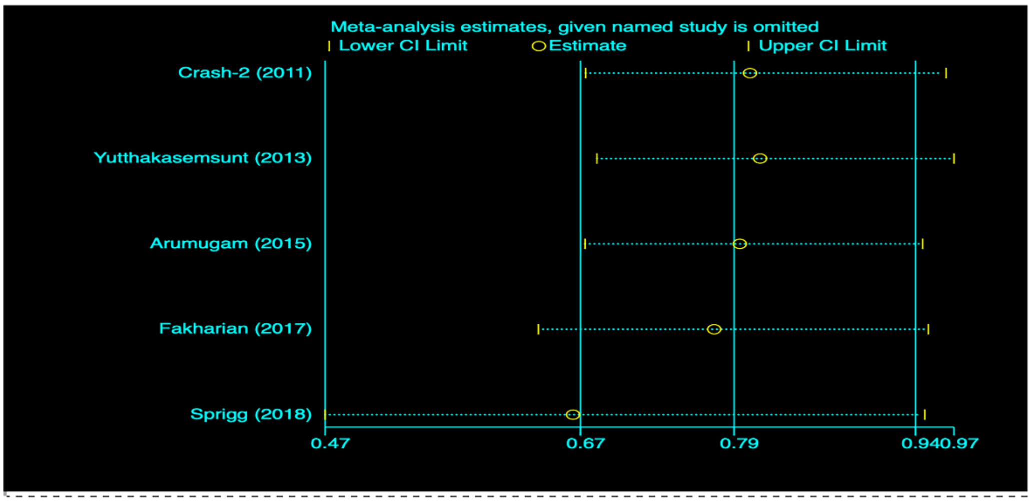

Study omitted	Estimate	[95% Conf.	Interval]
Crash-2 (2011)	.80741233	.67693955	.96303236
Yutthakasemsunt (2013)	.81540102	.68594509	.96928877
Arumugam (2015)	.79930443	.67650008	.94440138
Fakharian (2017)	.77896619	.63944143	.94893497
Sprigg (2018)	.66692144	.47013998	.94606769
Combined	.79471671	.67278404	.93874796

Figure S3. Influence analysis of tranexamic acid and the placebo treatments for hematoma expansion.

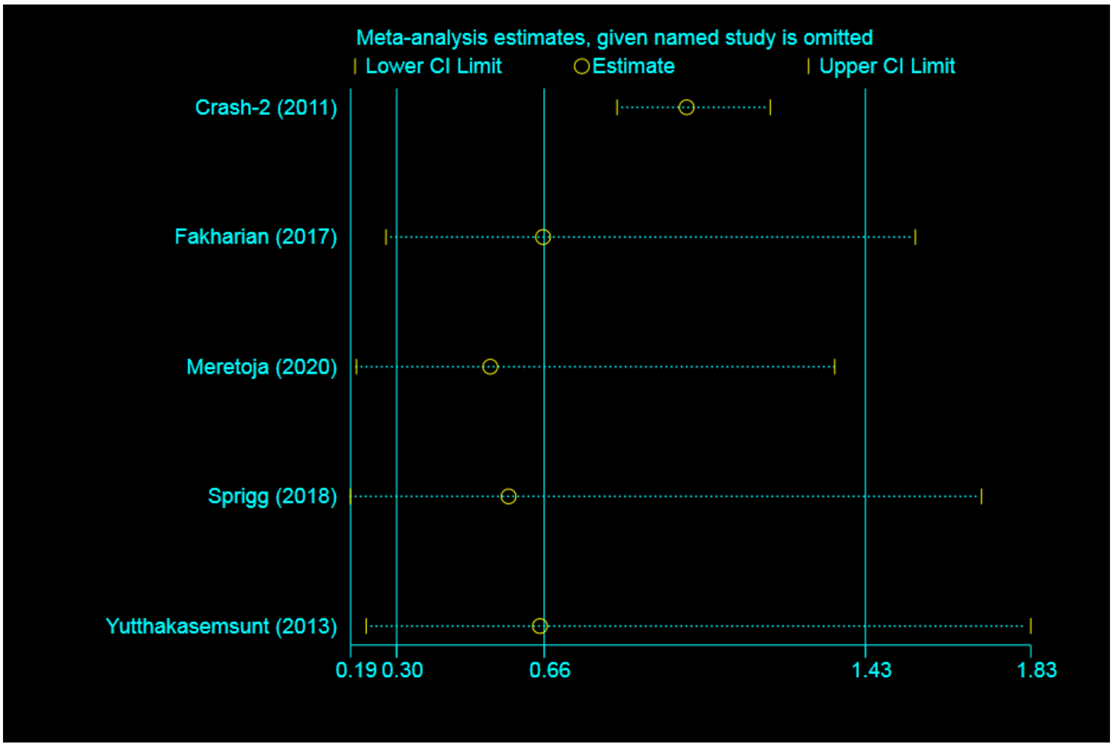

Figure S4. Influence analysis of tranexamic acid and the placebo treatments for the volume of the hemorrhagic lesion.

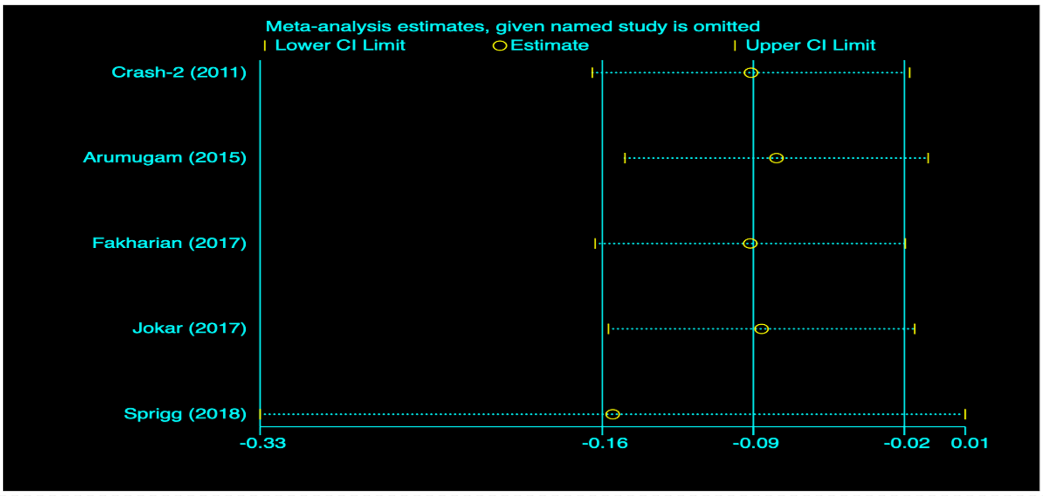

Study omitted	Estimate	[95% Conf.	Interval]
Crash-2 (2011)	-.09049452	-.16773909	-.01324994
Arumugam (2015)	-.07809751	-.15189402	-.004301
Fakharian (2017)	-.09087471	-.16636878	-.01538063
Jokar (2017)	-.08537356	-.15991367	-.01083345
Sprigg (2018)	-.15782766	-.32946712	.01381179
Combined	-.08935252	-.16284417	-.01586087

Figure S5. Influence analysis of tranexamic acid and the placebo treatments for the mortality.
